# Acute fiber supplementation with inulin-type fructans curbs appetite sensations: a randomized, double-blind, placebo-controlled study

**DOI:** 10.1080/16546628.2017.1341808

**Published:** 2017-07-02

**Authors:** Younis A. Salmean

**Affiliations:** ^a^ Department of Food Science and Nutrition, College of Life Sciences, Kuwait University, Kuwait

**Keywords:** Visual analogue scale, VAS, hunger, weight management, weight control, fermentable fiber, fiber

## Abstract

**Background:** Research points to a benefit of inulin fiber on appetite and weight regulation but results remain mixed. Objectives: To test the impact of 16 g/d of Inulin-type fructans (ITFs) on appetite and food intake in acute settings.

**Design:** Forty college age females received either a fiber drink with 16 g of ITFs in 330 ml water or placebo. On the 8^th^ day of the study, appetite sensations were assessed using visual analogue scale (VAS) along with food intake. Repeated-measures ANOVA were performed comparing VAS ratings during test day. Energy consumption was compared using paired t-tests. Significance was determined at p<0.05.

**Results:** On the 8^th^ day, the fiber group reported lower ratings for hunger, desire to eat, and prospective food consumption with significantly higher ratings for satisfaction and fullness. Subsequently, the fiber group consumed 21% less kcal from food at lunch (453 ± 47 kcal) compared to controls (571 ± 39 kcal) (p<0.05).

**Conclusions:** Consuming 16 g/d of ITFs in the morning for 7 days, and after an overnight fast, curbed appetite sensations and helped reduce food intake during lunch meal. These findings highlight the potential of using ITFs in weight management. Future studies should explore ITFs long term benefits.

## Introduction

Different types of fiber have different physical and chemical properties, all of which can affect digestion, appetite regulation, and energy consumption differently. Studies show that consumption of foods with a higher fiber content affects weight regulation [1,2], enhances satiety [3,4], and reduces energy intake [3,5]. In particular, there is growing interest in fermentable fibers, such as inulin-type fructans (ITFs), and the impact they have on the human body. ITFs are linear polysaccharides composed mainly of β-(2→1) fructosyl–fructose linkages [6]. They are practical to use as nutritional supplements because of their non-viscous and readily dissolvable nature, which makes them easy to prescribe for consumption. They are classified as prebiotic, stimulating the growth of bifidobacteria [7,8] and hence increasing the production of short-chain fatty acids (SCFAs).

Animal studies show that higher SCFA production increases levels of circulating appetite, inhibiting glucagon-like peptide-1 (GLP-1) [9–11] and peptide YY (PYY) [12,13]. Data from human studies show that consuming fermentable fibers, such as ITFs, can also increase concentrations of GLP-1 [4,10,14] and PYY [10,12,15], and reduce levels of ghrelin [12,16], a hormone that enhances appetite and leads to increased food intake [17].

Taking these findings together, one can postulate that ITFs are likely to affect appetite and satiety, and consequently energy intake. Results from the limited number of human studies available, however, are inconsistent. Karalus et al. [18] reported that 10 g of ITFs did not affect food intake or satiety sensations compared to controls in acute settings. Another acute study using a smaller dose of ITFs, 5–8 g/day in a beverage, failed to elicit any impact on satiety sensations, but resulted in reduced food intake in women only [19]. When looking at a higher dose, 21 g/day of ITFs for 12 weeks resulted in reduced body weight and reduced self-reported energy intake coupled with a positive impact on plasma satiety hormones [12]. Cani et al. [4] reported that 16 g/day of ITFs led to enhanced satiety and reduced energy consumption, while in a small (*n* = 10) short-term study, Cani et al. [15] reported that 16 g/day of a different ITF led to increased GLP-1 and PYY but failed to affect satiety ratings.

Collectively, these studies potentiate that ITF fibers may exert a desirable effect on appetite and/or food intake, but inconsistencies in findings warrant further investigation. Thus, and to contribute further to the understanding of the potential impact of ITF fibers on appetite, we carried out a randomized, double-blind, placebo-controlled study evaluating the impact of short-term supplementation of ITF fiber (16 g/day) on appetite sensations and subsequent food consumption in a homogeneous female population.

## Methods

### Subjects

Participants were recruited by flyers, posted advertisements on school boards, and by word of mouth across various Kuwait University campuses. Apparently healthy, college-age female participants aged 18–25 years were recruited for the study. Exclusion criteria included participants who could not adhere to the study protocol and those who were planning to start new dietary practices during the study duration. Forty participants were needed for the study, based on previously published sample size estimates for visual analogue scale (VAS) ratings [2], with an additional 25% in sample size to account for potential loss of participants during the study duration. We screened 55 participants for eligibility, 40 of whom met the inclusion criteria and were recruited. All participants were then randomly assigned to either the fiber or control group (simple randomization). All participants were informed about the details of the study, and provided their signed written consent. Of the 40 participants enrolled, 36 completed the study. This study was performed in accordance with the Declaration of Helsinki and approval for the study was granted by Kuwait University Research Sector.

### Study design

This was a randomized, double-blind, placebo-controlled, short intervention study. It consisted of a 1 week adaptation period and a testing day. The fiber used was Frutafit® IQ (kindly donated by Sensus, Roosendaal, the Netherlands), which is a native inulin/oligofructans with average chain monomer length of 8–13 and with excellent dispensability and wettability. During the study, 16 g of ITF fiber was provided daily to participants in the fiber group using an opaque water bottle with 330 ml of artificially flavored water (fiber group). Artificially flavored water in identical, opaque water bottles, devoid of fiber, was provided to the control group as a placebo. Study participants were instructed to consume the flavored water daily each morning between 7 and 8am with the breakfast meal. Potential adverse effects, including stool frequency, diarrhea, bloating and flatus, nausea, and stomach and intestinal discomfort, were assessed daily using a questionnaire. Participants were asked to fill out a daily VAS between 1 and 2pm and before lunch to assess the impact on appetite sensations in free-living conditions [20]. To reduce variability associated with free-living conditions, participants were instructed to consume the fiber, breakfast, and lunch, and to take the VAS at the specified hours. All questionnaires were collected together on the test day. In addition, and to correctly assess the impact on appetite sensations, VAS questionnaires were administered during the test day at set intervals in a controlled environment. Energy consumption was determined by pre- and post-weighing of breakfast and lunchtime meals.

### Weight

The body weight of the participants was measured while wearing regular clothes, without shoes, to the nearest ± 0.1 kg at the beginning and end of the study. Participants were asked to wear the same clothes during the end visit to reduce variability in weight associated with changing their attire. Height was measured to ± 0.25 cm at the beginning of the study using a wall-mounted stadiometer.

### Visual analogue scale

Participants were asked to fill out a 100 mm VAS for 7 consecutive days before typical lunch meals to examine the causal impact of consuming ITF fiber on perceived appetite sensations with and without fiber in free-living settings. In addition, VAS was administered during the test day, after an overnight fast, at set intervals after breakfast was finished (i.e. 20, 50, 95, 155, 200, 245 min post-breakfast). The VAS questions were ‘How strong is your desire to eat?’, ‘How hungry do you feel?’, ‘How full do you feel?’, ‘How satisfied do you feel?’, and ‘How much do you think you could eat right now?’, with terms anchored at either end of the 100 mm scale to indicate minimum or maximum response.

### Timeline protocol

Participants arrived on the test day having fasted overnight (no food or drink after midnight). Participants consumed the corresponding drink and waited for 15 min before breakfast was served. Participants were allowed 15 min to complete their meals. Once breakfast was finished, participants waited for 20 min before they filled out the first VAS to assess appetite-related sensations (20 min mark). Lunch was served at the 200 min mark and participants were allowed 25 min to finish their lunch and another 20 min before the last VAS was filled out.

### Meals

On the test day, two meals were provided for participants to consume: a breakfast consisting of 60 g of cereal (225 kcal) and 180 ml of 2% milk (72 kcal), and a lunch consisting of a chicken breast sandwich (425 g) with 25 g chips (134 kcal) and 300 ml of orange juice (150 kcal). All participants were allowed enough time to consume the corresponding meals until they felt comfortably full. Foods and drinks were weighed pre- and post-meal, separately, to determine energy consumption.

### Statistical analysis

All data are expressed as mean ± SEM. Mean VAS ratings for each group were compared using the paired Student’s *t* test during the adaptation period and for energy intake. Repeated-measures analysis of variance (ANOVA) was performed to determine treatment effects on VAS ratings between the two groups. Statistical significance was set at *p* < 0.05. Statistical analyses were carried out using SPSS version 20 (IBM Corp, Armonk, NY, USA).

## Results

### Participants’ characteristics

Of the 40 participants, four did not complete the study for personal reasons. There was no difference in age between the control group (19.6 ± 0.48 years) and the fiber group (19.8 ± 0.33 years). There was no difference in initial and end of study body weights between the two groups. The control group, however, gained weight at the end of the study compared to its baseline weight, from 60.1 ± 2.7 kg to 60.7 ± 2.6 kg (*p* = 0.037), while the fiber group did not see a significant weight increase.

### Anticipated intestinal adverse effects

There was no difference between the two groups in reported bowel movements or the reported incidence of diarrhea, nausea, or intestinal discomfort. The fiber group, however, had a 20% increase in reported incidences of bloating and flatus (*p* = 0.029).

### Daily VAS ratings

During the adaptation period, the fiber group reported lower ratings for desire to eat, hunger, and prospective food consumption, with higher ratings for fullness compared to the control group (Table 1).

**Table 1.** Pre-lunch visual analogue scale (VAS) ratings during the 7 day adaptation period.

VAS question	Control (*n* = 19)	Fiber (*n* = 17)	*p*
How strong is your desire to eat?	64 ± 2.2	52 ± 2.9	0.002
How hungry do you feel?	65 ± 2.1	55 ± 2.8	0.008
How full do you feel?	32 ± 2.0	37 ± 2.4	< 0.001
How satisfied do you feel?	36 ± 2.0	35 ± 2.2	NS
How much do you think you could eat right now?	68 ± 1.9	55 ± 2.4	0.048

Data are shown as mean ± SEM.

NS, not significant.

### VAS appetite profile changes during the test day

**Figure 1.**
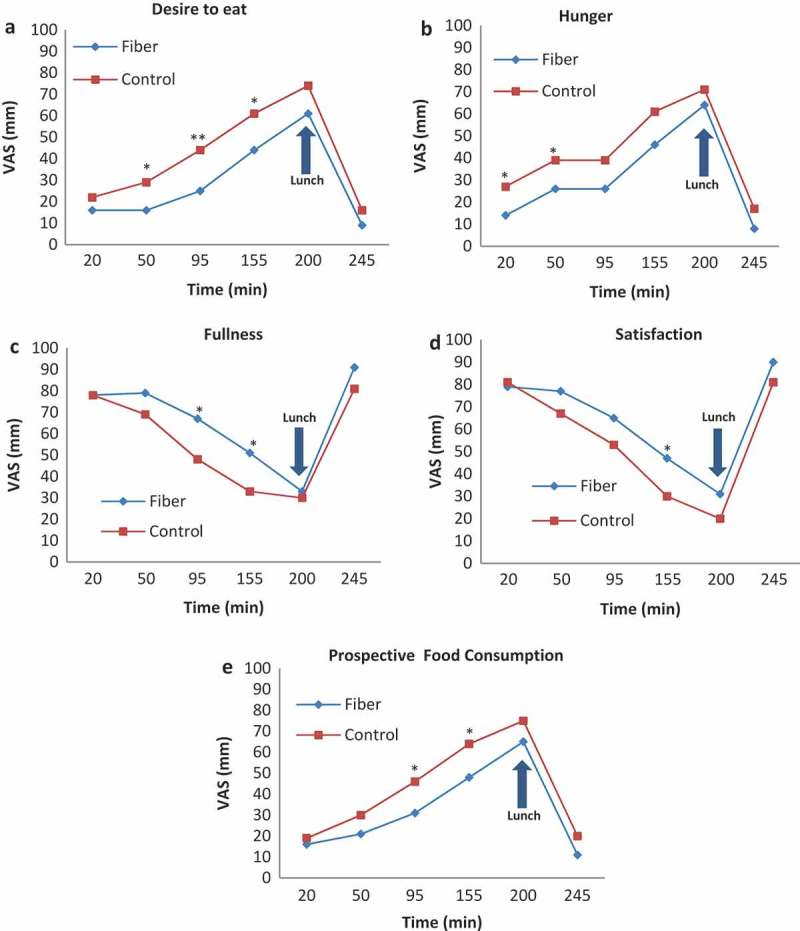
Changes in visual analogue scale (VAS) domains over 245 min post-breakfast. VAS ratings are shown for desire to eat, hunger, fullness, satisfaction, and prospective food consumption over 245 min post-breakfast. Intervals are in minutes and indicate time of VAS administration. Values are mean ± SEM; fiber *n* = 17, control *n* = 19. Significance at *p < 0.05,**p < 0.001.

The control group had a significantly higher ‘desire to eat’ 50 min post-breakfast (*p* = 0.05) and this significance remained at 95 min (*p* = 0.007) and 155 min (*p* = 0.041) post-breakfast (Figure 1). The control group also reported higher ‘hunger’ ratings 20 min (*p* = 0.044) and 50 min (*p* = 0.042) post-breakfast. For ‘prospective food consumption’, the control group reported higher ratings at 95 min (0.034) and 155 min (*p* = 0.031) post-breakfast, with lower ‘satisfaction’ at 155 min (*p* = 0.027) post-breakfast. The fiber group reported significantly higher ‘fullness’ at 95 min (*p* = 0.037) and 155 min (*p* = 0.021).

### Energy intake

The control and fiber groups consumed the same amount of energy at breakfast on the test day (235 ± 18 kcal and 230 ± 16 kcal, *p* > 0.05), respectively. The total energy consumed at lunch by the fiber group was 554 ± 53 kcal compared to 666 ± 45 kcal consumed by the control group (*p* = 0.056). The fiber group consumed significantly less energy from foods at lunch compared to the control group (453 ± 47 kcal vs 571 ± 39 kcal, *p* = 0.031). There was no significant difference in the energy consumed from drinks at lunch.

## Discussion

As reviewed before, previous studies suggest that fiber can affect hunger and/or satiety favorably, leading to reduced energy intake [21]. Several animal studies have suggested that consumption of fermentable fibers can increase GLP-1 and proglucagon expression and improve glucose homeostasis [9,22,23]. However, to our knowledge, very few, mostly inhomogeneous studies with variable fiber doses have investigated the impact of ITFs or inulin supplementation on appetite, hunger sensations, and food intake in the adult population. In our study, when participants consumed 16 g/day of ITFs in the morning, the average ratings in their ‘desire to eat’, ‘hunger’, and ‘prospective food consumption’ were significantly lower compared to the control group. In addition, the fiber group reported higher ‘fullness’ ratings just before lunch, suggesting a potential impact from the fiber. However, because the adaptation period was a free-living phase, it is difficult to conclude that the observed benefits, although suggested, are due solely to the ITFs since the amount of food consumed from breakfast to lunch is unknown. However, looking at the data from the test day, where settings and food intake were controlled, it appears that consuming 16 g of ITFs in the morning lowered the desire to eat, hunger, and the interest in food consumption, and enhanced fullness and satisfaction for much longer compared to placebo. This is consistent with the findings of Cani et al. [4], who found that 16 g of ITFs promoted satiety in healthy humans.

The impact on appetite of the participants was due to ITF consumption since both groups consumed equal amounts of energy at breakfast (235 ± 18 kcal vs 230 ± 16 kcal) and were housed in exact conditions with minimal physical disturbances. The residual impact of the ITFs on appetite sensations may explain why the fiber group consumed 21% fewer calories from food at lunch, since most sensations were favorably different until 155 min post-breakfast, and shortly before lunch was served at 200 min. It is worth noting that the reduced consumption of food by the fiber group meant that they had more time to interact at the table during lunch, which may have led to a pattern of increased liquid consumption, a naturally anticipated response when liquids accompany meals. It would have been more ideal to provide water in place of juice, considering that liquid calories are less likely to elicit a precise dietary compensation response because swallowing does not trigger the internal satiety signal that masticating does [24].

One reason for previous studies failing to show a positive impact of fiber supplementation on VAS domains could be the use of inhomogeneous subject populations, in particular, a wider physiological age difference. Hess et al. [19], Harrold et al. [25], and Verhoef et al. [26] included subjects with age ranges from 18–65, 1–64, and 20–60 years, respectively. The innate physiological response and magnitude of appetite and energy regulation complexes for older and younger people can be quite different. Anorexigenic signals in older adults prevail over orexigenic signals, contributing to prolonged satiety and inhibition of hunger [27], which can easily affect VAS reporting in a mixed population of wide physiological age ranges. It is, therefore, suggested that more homogeneous subject populations be used when investigating the impact of fiber on appetite and energy regulation [28]. We designed our study to be practically homogeneous; thus, we enrolled college-age females, which may explain the agreement found in our study with that of Cani et al. [4], where fiber intake had a significant impact on satiety in 21–39-year-old participants.

Another possible reason for the inconsistency of the results of previous studies may be the different doses of ITFs used. The study by Karalus et al. [18] reported no significant benefits on appetite ratings or weight of supplementing the diet with 10 g ITF fiber. The likely explanation for this is the low fiber doses used in the study. The 10 g dose of ITFs is not likely to produce any marked impact on appetite and hunger sensations [29]. The 16 g of inulin used in our study is likely to be an effective dose to produce meaningful change in appetite sensations, as this amount has been shown to produce favorable changes in appetite sensations [4], and in appetite-related hormones and peptides [26].

Our data suggest an impact on weight in the short term. After a week of supplementation, the fiber group saw no significant increase in body weight compared to its baseline, but the control group had a significant increase from its baseline. This is consistent with the findings of Parnell and Reimer [12], where supplementation with ITFs resulted in a significant reduction in weight in the fiber group while the control group experienced a significant weight gain.

Participants consuming the fiber reported a higher incidence of bloating and flatulence, which was anticipated [30]. Although the incidence was higher among fiber consumers, the fiber was tolerated as no dropouts were reported as a result of supplement use.

### Limitations of the study

The lack of control for enrolment in relation to menstrual cycle may have been a limitation owing to the impact that the menstrual cycle has on appetite hormones [31] and weight fluctuations. Future studies should consider scheduling enrolment within the first 14 days of each participant’s monthly menstrual cycle (follicular phase) to increase consistency and avoid unwanted fluctuations in hormones that may lead to changes in appetite sensations and energy intake. Finally, the study participants consisted of only college-age females, with a narrow age range, and therefore the results may not be generalizable.

## Conclusion

In conclusion, dietary supplementation with 16 g/day of ITF fiber in the morning was found to reduce hunger, desire to eat, and prospective food consumption, and to increase fullness and satiety in acute settings, leading to reduced food intake at lunch. These results suggest that ITF fiber is potentially a useful adjunct dietary supplement for curbing appetite and possibly aiding weight management.

## References

[1] Slavin JL. Dietary fiber and body weight. Nutrition. 2005;21(3):411–6. 15797686 10.1016/j.nut.2004.08.018

[2] Pereira MA, Ludwig DS Dietary fiber and body-weight regulation. Observations and mechanisms. Pediatr Clin North Am. 2001;48(4):969–980. 11494646 10.1016/s0031-3955(05)70351-5

[3] Hull S, Re R, Tiihonen K, et al Consuming polydextrose in a mid-morning snack increases acute satiety measurements and reduces subsequent energy intake at lunch in healthy human subjects. Appetite. 2012;59(3):706–712. 22885981 10.1016/j.appet.2012.08.004

[4] Cani PD, Joly E, Horsmans Y, et al Oligofructose promotes satiety in healthy human: a pilot study. Eur J Clin Nutr. 2006;60(5):567–572. 16340949 10.1038/sj.ejcn.1602350

[5] Pelkman CL, Navia JL, Miller AE, et al Novel calcium-gelled, alginate-pectin beverage reduced energy intake in nondieting overweight and obese women: interactions with dietary restraint status. Am J Clin Nutr. 2007;86(6):1595–1602. 18065575 10.1093/ajcn/86.5.1595

[6] Liu J, Waterhouse AL, Chatterton NJ Proton and carbon NMR chemical-shift assignments for [beta-D-Fru f-(2→1)]3-(2≤≥1)-alpha-D-Glc p (nystose) and [beta-D-Fru f-(2→1)]4-(2≤≥1)-alpha-D-Glc p (1,1,1-kestopentaose) from two-dimensional NMR spectral measurements. Carbohydr Res. 1993;245(1):11–19. 8358742 10.1016/0008-6215(93)80056-k

[7] Bouhnik Y, Flourie B, Riottot M, et al Effects of fructo-oligosaccharides ingestion on fecal bifidobacteria and selected metabolic indexes of colon carcinogenesis in healthy humans. Nutr Cancer. 1996;26(1):21–29. 8844718 10.1080/01635589609514459

[8] Kleessen B, Hartmann L, Blaut M Oligofructose and long-chain inulin: influence on the gut microbial ecology of rats associated with a human faecal flora. Br J Nutr. 2001;86(2):291–300. 11502244 10.1079/bjn2001403

[9] Massimino SP, McBurney MI, Field CJ, et al Fermentable dietary fiber increases GLP-1 secretion and improves glucose homeostasis despite increased intestinal glucose transport capacity in healthy dogs. J Nutr. 1998;128(10):1786–1793. 9772150 10.1093/jn/128.10.1786

[10] Cani PD, Dewever C, Delzenne NM Inulin-type fructans modulate gastrointestinal peptides involved in appetite regulation (glucagon-like peptide-1 and ghrelin) in rats. Br J Nutr. 2004;92(3):521–526. 15469657 10.1079/bjn20041225

[11] Reimer RA, McBurney MI Dietary fiber modulates intestinal proglucagon messenger ribonucleic acid and postprandial secretion of glucagon-like peptide-1 and insulin in rats. Endocrinology. 1996;137(9):3948–3956. 8756571 10.1210/endo.137.9.8756571

[12] Parnell JA, Reimer RA Weight loss during oligofructose supplementation is associated with decreased ghrelin and increased peptide YY in overweight and obese adults. Am J Clin Nutr. 2009;89(6):1751–1759. 19386741 10.3945/ajcn.2009.27465 PMC3827013

[13] Delzenne NM, Cani PD, Daubioul C, et al Impact of inulin and oligofructose on gastrointestinal peptides. Br J Nutr. 2005;93(Suppl 1):S157–S161. 15877889 10.1079/bjn20041342

[14] Piche T, des Varannes SB, Sacher-Huvelin S, et al Colonic fermentation influences lower esophageal sphincter function in gastroesophageal reflux disease. Gastroenterology. 2003;124(4):894–902. 12671885 10.1053/gast.2003.50159

[15] Cani PD, Lecourt E, Dewulf EM, et al Gut microbiota fermentation of prebiotics increases satietogenic and incretin gut peptide production with consequences for appetite sensation and glucose response after a meal. Am J Clin Nutr. 2009;90(5):1236–1243. 19776140 10.3945/ajcn.2009.28095

[16] Tarini J, Wolever TM The fermentable fibre inulin increases postprandial serum short-chain fatty acids and reduces free-fatty acids and ghrelin in healthy subjects. Appl Physiol Nutr Metab. 2010;35(1):9–16. 20130660 10.1139/H09-119

[17] Wren AM, Seal LJ, Cohen MA, et al Ghrelin enhances appetite and increases food intake in humans. J Clin Endocrinol Metab. 2001;86(12):5992. 11739476 10.1210/jcem.86.12.8111

[18] Karalus M, Clark M, Greaves KA, et al Fermentable fibers do not affect satiety or food intake by women who do not practice restrained eating. J Acad Nutr Diet. 2012;112(9):1356–1362. 22771185 10.1016/j.jand.2012.05.022

[19] Hess JR, Birkett AM, Thomas W, et al Effects of short-chain fructooligosaccharides on satiety responses in healthy men and women. Appetite. 2011;56(1):128–134. 21146572 10.1016/j.appet.2010.12.005

[20] Flint A, Raben A, Blundell JE, et al Reproducibility, power and validity of visual analogue scales in assessment of appetite sensations in single test meal studies. Int J Obes Relat Metab Disord. 2000;24(1):38–48. 10702749 10.1038/sj.ijo.0801083

[21] Howarth NC, Saltzman E, Roberts SB Dietary fiber and weight regulation. Nutr Rev. 2001;59(5):129–139. 11396693 10.1111/j.1753-4887.2001.tb07001.x

[22] Gee JM, Lee-Finglas W, Wortley GW, et al Fermentable carbohydrates elevate plasma enteroglucagon but high viscosity is also necessary to stimulate small bowel mucosal cell proliferation in rats. J Nutr. 1996;126(2):373–379. 8632208 10.1093/jn/126.2.373

[23] Reimer RA, Thomson AB, Rajotte RV, et al A physiological level of rhubarb fiber increases proglucagon gene expression and modulates intestinal glucose uptake in rats. J Nutr. 1997;127(10):1923–1928. 9311946 10.1093/jn/127.10.1923

[24] Haber GB, Heaton KW, Murphy D, et al Depletion and disruption of dietary fibre. Effects on satiety, plasma-glucose, and serum-insulin. Lancet. 1977;2(8040):679–682. 71495 10.1016/s0140-6736(77)90494-9

[25] Harrold JA, Hughes GM, O’Shiel K, et al Acute effects of a herb extract formulation and inulin fibre on appetite, energy intake and food choice. Appetite. 2013;62:84–90. 23207186 10.1016/j.appet.2012.11.018

[26] Verhoef SP, Meyer D, Westerterp KR Effects of oligofructose on appetite profile, glucagon-like peptide 1 and peptide YY3-36 concentrations and energy intake. Br J Nutr. 2011;106(11):1757–1762. 21679485 10.1017/S0007114511002194

[27] Di Francesco V, Zamboni M, Zoico E, et al Unbalanced serum leptin and ghrelin dynamics prolong postprandial satiety and inhibit hunger in healthy elderly: another reason for the “anorexia of aging”. Am J Clin Nutr. 2006;83(5):1149–1152. 16685059 10.1093/ajcn/83.5.1149

[28] Howarth NC, Saltzman E, McCrory MA, et al Fermentable and nonfermentable fiber supplements did not alter hunger, satiety or body weight in a pilot study of men and women consuming self-selected diets. J Nutr. 2003;133(10):3141–3144. 14519798 10.1093/jn/133.10.3141

[29] Tovar AR, Caamano Mdel C, Garcia-Padilla S, et al The inclusion of a partial meal replacement with or without inulin to a calorie restricted diet contributes to reach recommended intakes of micronutrients and decrease plasma triglycerides: a randomized clinical trial in obese Mexican women. Nutr J. 2012;11:44. 22703579 10.1186/1475-2891-11-44 PMC3489692

[30] Liber A, Szajewska H Effects of inulin-type fructans on appetite, energy intake, and body weight in children and adults: systematic review of randomized controlled trials. Ann Nutr Metab. 2013;63(1–2):42–54. 10.1159/000350312 23887189

[31] Sramkova M, Duskova M, Vitku J, et al Levels of adipokines and some steroids during the menstrual cycle. Physiol Res. 2015;64 Suppl 2:S147–S154. 26680475 10.33549/physiolres.933116

